# A practical lead exchange technique without additional venous puncture in the era of conduction system pacing

**DOI:** 10.1016/j.hroo.2026.05.009

**Published:** 2026-05-21

**Authors:** Yoshimi Onishi, Mina Utsunomiya, Rihito Mitsuhashi, Miyu Tsunoda, Yoshikazu Suzuki, Saori Chino, Hiroto Sugiyama, Yuya Nakamura, Masaaki Kurata, Yoshitaka Iso, Hiroshi Suzuki

**Affiliations:** 1 Division of Cardiology, Department of Medicine, Showa Medical University Fujigaoka Hospital, Yokohama, Kanagawa, Japan; 2 Division of Cardiology, Department of Medicine, Showa Medical University, Shinagawa, Tokyo, Japan; 3 Department of Clinical Engineering, Showa Medical University Fujigaoka Hospital, Yokohama, Kanagawa, Japan

**Keywords:** Lead exchange, Conduction system pacing, Guidewire, Lead, Venous access

**Key Findings**

▪ Upgrades from right ventricular apical pacing to conduction system pacing may require lead replacement, but additional venous puncture can introduce procedural risk. This report presents a practical technique that preserves venous access without additional puncture.
▪ The technique uses a retained intravenous cannula sheath placed between the outer insulation and the conductor coil of a nonadherent transvenous lead, allowing subsequent guidewire insertion and delivery of a long guiding catheter.
▪ A practical advantage of this approach is that it enables direct transition to long delivery systems and may also allow temporary unipolar backup pacing when structurally appropriate leads are used.
▪ Although 18-G cannula retention was difficult or unstable in some lead models, all evaluated leads accommodated a 20-G intravenous cannula; when a 20-G cannula was required, the technique could be completed with a 0.025-in guidewire.

As upgrades from right ventricular apical pacing to conduction system pacing (CSP) become more common, lead replacement is often assumed to require additional venous puncture for delivery of a long guiding catheter. Here, we describe a practical technique for nonadherent transvenous lead exchange using a retained intravenous (IV) cannula sheath, with assessment of lead-specific feasibility across current lead models.

The step-by-step procedure was demonstrated using a transparent acrylic model to illustrate the key maneuvers of the technique: prerequisite: release the suture sleeve and confirm lead mobility.

*Step 1:* Puncture the outer insulation of the lead near the venous entry site using an 18-G IV cannula and advance the cannula sheath approximately 2–3 mm between the outer insulation and the conductor coil (Figure 1A).

**Figure 1:**
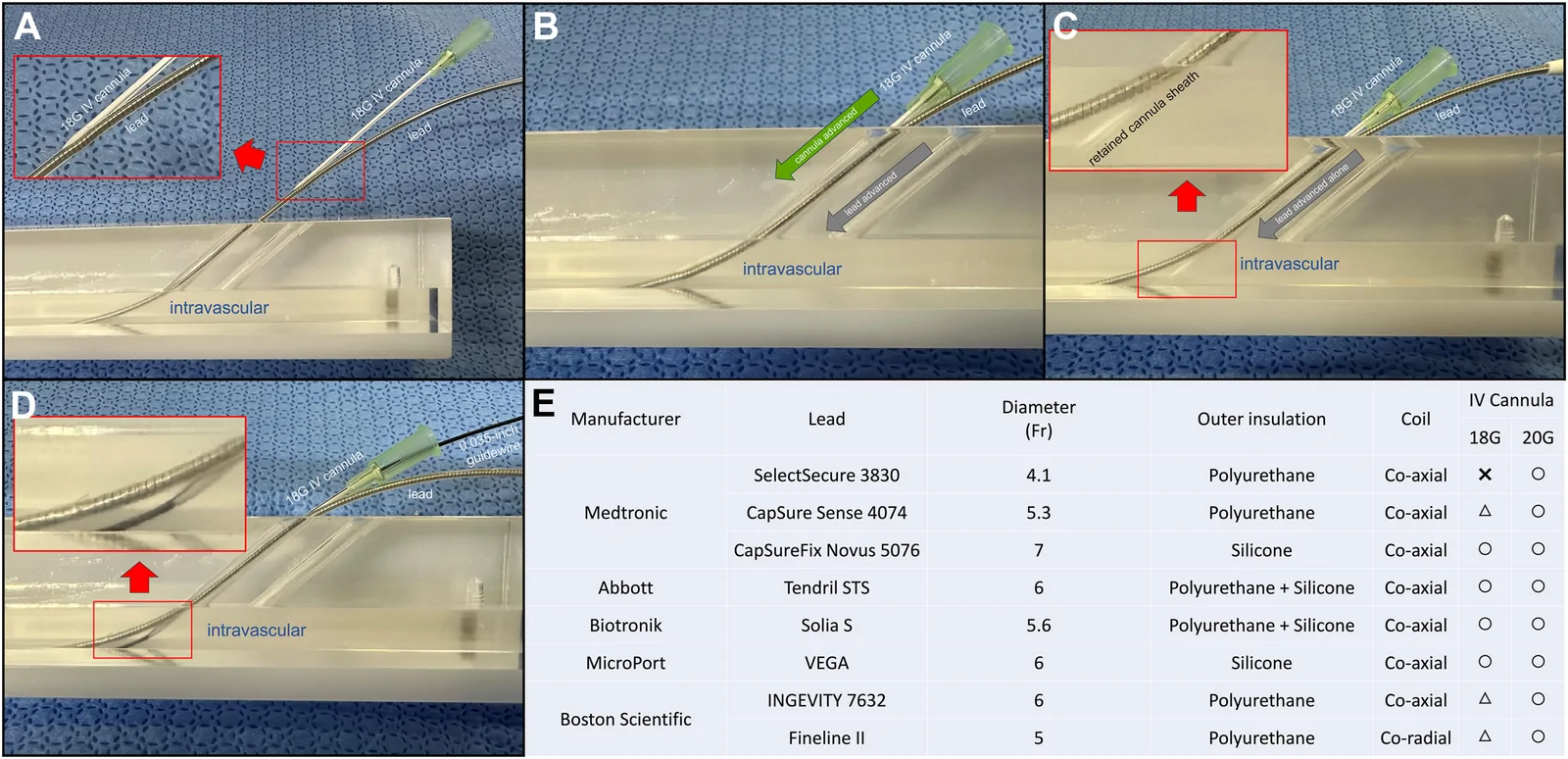
Stepwise demonstration of the technique using an acrylic model (**A–D**) and in vitro lead applicability across evaluated current lead models (**E**). The *open circle* denotes successful in all 3 attempts; open triangle, successful in 1 or 2 of 3 attempts; and cross, unsuccessful in all 3 attempts. IV = intravenous.
*Step 2:* Advance the lead together with the IV cannula sheath into the vein until the cannula hub reaches the venous entry site (Figure 1B).
*Step 3:* Advance the lead alone further into the vein. This allows the IV cannula sheath to separate from the lead and remain within the vein as retained venous access (Figure 1C).
*Step 4*: Confirm intravascular placement by backflow of blood through the cannula. Contrast injection may be used if needed. After confirmation of intravascular retention, insert a 0.035-in guidewire through the retained IV cannula sheath (Figure 1D).
*Step 5:* If intrinsic rhythm is present, the lead is removed and the CSP guiding catheter is advanced over the wire. If intrinsic rhythm is absent, a coaxial lead can be kept in place without prior removal and used for temporary unipolar backup pacing, because the cathodal coil remains intact despite insulation puncture. Even with the lead retained, the CSP guiding catheter can then be advanced over the already-positioned guidewire alongside the retained lead.

In vitro puncture feasibility and cannula retention across the current lead models evaluated in this study are summarized in Figure 1E. Each lead was tested in 3 attempts. In all evaluated leads, cannula insertion was completed within 1 minute.

Antonelli et al^1^ reported a retained-wire technique in which the lead insulation is opened, a vein lifter is inserted between the lead and its encapsulating sleeve, and a guidewire is advanced into this plane before the lead and wire are advanced together into the vein. Because this technique requires wire insertion into a narrow plane using a relatively bulky vein lifter, wire advancement may be technically demanding, and a spring-type J guidewire cannot be used initially. By contrast, in the present technique, the guidewire is inserted only after stable retention of the cannula sheath, allowing greater flexibility in guidewire selection and a simpler workflow.

Mendenhall^2^ described a sheath-based suture-traction technique in which the lead is drawn through a nonsplittable sheath to preserve venous access during lead replacement. However, its procedural concept differs from the present technique. Because access is retained through an outer sheath, placement of a long CSP guiding catheter requires an additional sheath-based step, which may necessitate use of a larger outer sheath and may increase the risk of postoperative bleeding. In addition, because the lead is cut in the Mendenhall technique, temporary unipolar backup pacing is not possible. More fundamentally, the present technique provides long guidewire access without additional venous puncture. Once long guidewire access has been established, long guiding catheters can be introduced directly not only for CSP but also for procedures such as left ventricular lead placement or Bachmann bundle pacing. The same approach may be useful during initial implantation when lead dislodgment after peel-away necessitates reinsertion of a long delivery system.

An additional strength of the technique is its applicability across current lead models. Feasibility depended mainly on lead diameter and outer insulation characteristics. Retention of an 18-G IV cannula was difficult in SelectSecure 3830 (Medtronic) and unstable in leads with polyurethane-only outer insulation, whereas a 20-G cannula provided stable retention in both settings and could be used with a 0.025-in guidewire when required. Fineline II (Boston Scientific) was the only evaluated lead with a coradial design, and temporary unipolar backup pacing was considered unsuitable because needle puncture may injure the cathodal coil.

In conclusion, we describe a practical lead exchange technique in which an IV cannula is inserted into a nonadherent transvenous lead to preserve venous access without additional venous puncture. The technique enables subsequent delivery of a CSP guiding catheter, making it a practical option in the CSP era.

## Disclosures

The authors have no conflicts of interest to disclose.
